# Immunopathology of lung transplantation: from infection to rejection and vice versa

**DOI:** 10.3389/fimmu.2024.1433469

**Published:** 2024-09-02

**Authors:** Ilaria Righi, Ivan Barone, Lorenzo Rosso, Letizia Corinna Morlacchi, Valeria Rossetti, Giovanni Caffarena, Fiona Limanaqi, Alessandro Palleschi, Mario Clerici, Daria Trabattoni

**Affiliations:** ^1^ Thoracic Surgery and Lung Transplant Unit, Fondazione IRCCS Ca’ Granda Ospedale Maggiore Policlinico, Milan, Italy; ^2^ Respiratory Unit and Cystic Fibrosis Adult Center, Fondazione IRCCS Ca’ Granda Ospedale Maggiore Policlinico, Milan, Italy; ^3^ Department of Pathophysiology and Transplantation, University of Milan, Milan, Italy; ^4^ Department of Thoracic Surgery, IEO, European Institute of Oncology IRCCS, Milan, Italy; ^5^ Department of Biomedical and Clinical Sciences (DIBIC), University of Milan, Milan, Italy; ^6^ Fondazione Don C. Gnocchi IRCCS, Milan, Italy

**Keywords:** lung transplantation, infection, rejection, immune tolerance, immunosuppression

## Abstract

Lung transplantation offers a lifesaving option for patients with end-stage lung disease, but it is marred by a high risk of post-transplant infections, particularly involving multidrug-resistant bacteria, Cytomegalovirus, and fungal pathogens. This elevated infection rate, the highest among solid organ transplants, poses a significant challenge for clinicians, particularly within the first year post-transplantation, where infections are the leading cause of mortality. The direct exposure of lung allografts to the external environment exacerbates this vulnerability leading to constant immune stimulation and consequently to an elevated risk of triggering alloimmune responses to the lung allograft. The necessity of prolonged immunosuppression to prevent allograft rejection further complicates patient management by increasing susceptibility to infections and neoplasms, and complicating the differentiation between rejection and infection, which require diametrically opposed management strategies. This review explores the intricate balance between preventing allograft rejection and managing the heightened infection risk in lung transplant recipients.

## Introduction

**Figure 1:**
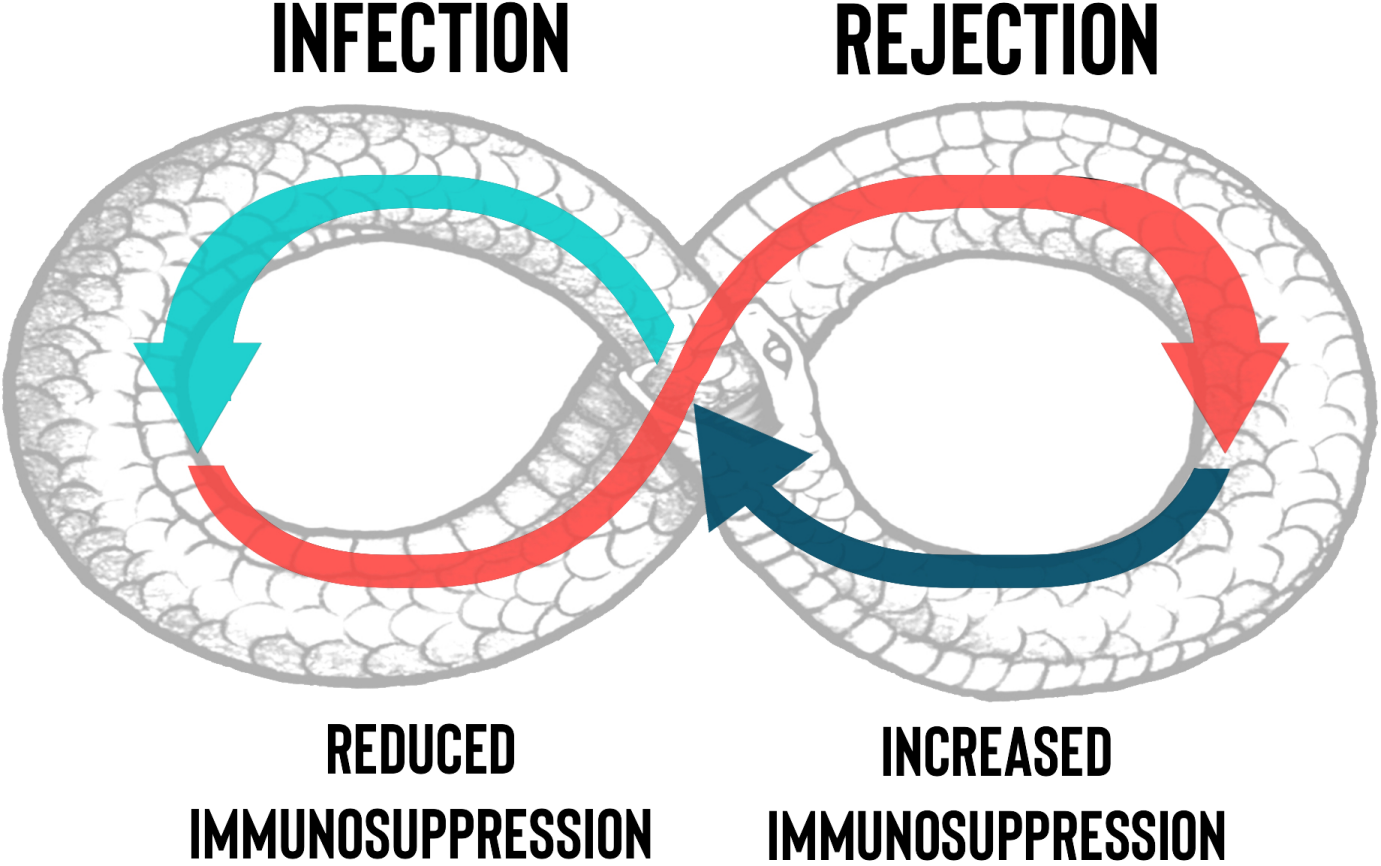
Management of the net state of immunosuppression in lung transplantation. The necessity by clinicians to decrease the immunosuppressants dosage, due to the onset of an infection or drugs’ side effects, could trigger rejection with a “snake who bites its own tail” effect. The figure describes at a glance the difficult to obtain balance between the risk of infection and rejection in lung transplant.

Compared with other solid organ transplants (SOT), lung transplantation (LuTx) has a higher post-transplant infection rate, which is characterized by a higher frequency of multidrug-resistant bacterial infections, a heightened burden of cytomegalovirus (CMV) infection, and a greater invasive fungal infection rate (1–3). In fact, differently from other SOT, lung allografts are in direct communication with the external environment, thus being constantly exposed to air pollutants and pathogens. The continuous contact with these agents, the impairing of muco-ciliary clearance caused by the denervation of the transplanted lung and the presence of bronchial anastomoses, as well as the presence of large numbers of donor-derived dendritic cells, leads to constant immune stimulation (4). This likely favors direct and indirect recognition of antigens expressed on the transplanted lungs by host alloreactive T lymphocytes, triggering alloimmune responses against the graft (5). Preventing lung allograft rejection requires suppression of both cell-mediated and humoral responses, which can be achieved by potent immune suppressants, initiated shorty before surgery and maintained throughout the recipient’s life (6). Unfortunately, prolonged immunosuppression often results in the generation of long-term toxicities and confers an augmented susceptibility to infections and neoplasms (7, 8). Moreover, differential diagnosis between lung allograft rejection and infection can be challenging and, most importantly, therapeutic approaches for both conditions differ much; the first requires maximization of immunosuppression including high-dose glucocorticoids, whereas the second needs targeted anti-microbial therapy plus tapering of immunosuppressive drugs (9, 10). The objective of this review is to elucidate the complex interplay between infection and rejection in LuTx and the clinical conundrum posed by the necessity of balancing risks for graft rejection against risks for infection, graphically represented as a snake that bites its own tail (**Figure 1**).

## Infection of the lung allograft as a trigger for rejection

In LuTx, infections can be derived from the donor, be reactivated latent infections of the recipient, or be newly acquired (11). Bacterial pneumonia and bronchitis are the most common, but infections caused by fungi, CMV, other viruses, and mycobacteria collectively contribute to the burden (12–14). Infection of the lung allograft can be the trigger of immunological interactions between donor and recipient, which is the basis of the processes that then lead to acute and chronic rejection (15, 16). The most accepted hypothesis is that tissue damage to the lung allograft induces the production of pathogen-related molecules, capable of abnormally stimulating innate and adaptive immune responses (17).

**Figure 2:**
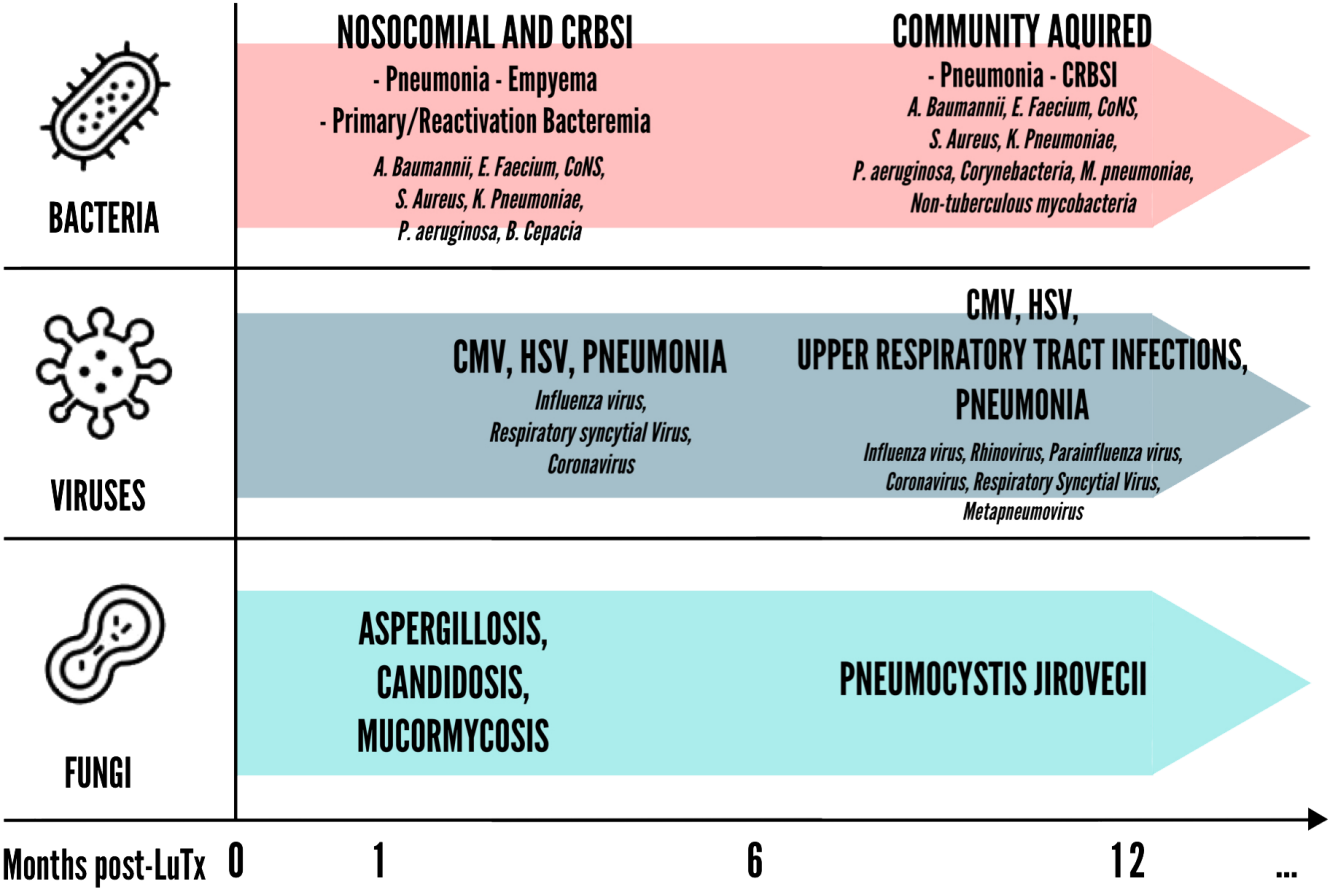
Timing of bacterial, viral and fungal infections in lung post-transplantation. CMV, Cytomegalovirus; CoNS, Coagulase-negative staphylococci; CRBSI, Catheter-related bloodstream infection; HSV, Herpes simplex virus;.

Furthermore, it seems that the time from LuTx at which infection occurs influences the quality of the ensuing immune response. Infections occurring before transplantation stimulate heterologous immunity, a process resulting from previous immunological exposure and mediated by memory cross-reactive T cells that may influence future immune response to unrelated pathogens (18, 19). This phenomenon is supported by the presence of environmentally primed T cells in the recipient that cross-react with donor antigens. Studies have shown that these pre-transplant donor-specific T cells, which can be identified by their IFN-γ production, correlate with a higher post-transplant risk of acute rejection episodes (20). The environmental antigen exposure of the recipient is independent of HLA mismatches between donor and recipient, highlighting the role of pre-existing immune memory in rejection risk. On the other hand, infections occurring late after transplantation may elicit pro-inflammatory signals, such as IL-17 and IFN-γ, which activate resting T cells and favor their escape from immunosuppression, thereby precipitating rejection (21). Notably, a critical threshold of memory T cells, particularly CD8+ central memory T cells, is needed to promote rejection. These cells are primarily responsible for the strong immune responses seen in these scenarios and can significantly hinder tolerance induction (19). For these reasons, infections in LuTx are categorized differently whether they occur less than one month after LuTx, between one to six months, or after more than six months from LuTx (**Figure 2**).

### Bacterial infections

Bacterial infections are the most common early infectious complications after LuTx. In addition to pneumonia, LTR are at heightened risk of other types of infection, including empyema, bloodstream infection, and wound infection (22, 23). The pathogens most frequently responsible for pneumonia in LTR are *Pseudomonas aeruginosa*, *Staphylococcus aureus* and *Enterobacteriaceae* (24). *Pseudomonas aeruginosa*, in particular, is able to trigger potent innate immune responses mediated by the granulocyte-colony stimulating factor (G-CSF), promoting allograft neutrophil infiltration. Additionally, *Pseudomonas aeruginosa* infection induces allograft-infiltrating neutrophils to upregulate B7 molecules (CD80 and CD86), which may enhance alloantigen-specific T cell responses through B7 costimulation. These findings underscore the potential of *Pseudomonas aeruginosa* to exacerbate alloimmune responses, potentially contributing to antibody-mediated rejection (AMR) and chronic lung allograft dysfunction (CLAD) post-transplantation (25). It has also been demonstrated that the activation of toll-like receptors by bacterial colonization of the donor airways prevents the induction of lung allograft tolerance through a process mediated by recipient-derived monocytes, thus facilitating allograft rejection (26). In murine models in which lung tolerance was established, neutrophil B7 expression induced by *Pseudomonas aeruginosa* invalidated tolerance through promoting T cell trans-costimulation, showing again the importance of toll-like receptors in regulating organ tolerance (27).

### Cytomegalovirus infection

CMV is the second most common cause of infection in LTR after bacterial pneumonia (28). CMV is known for its capability to establish lifelong latent infections. Its effects on T-cells are significant and multifaceted, impacting both the immune response to the virus itself and the broader immune system. CMV infection induces a robust and sustained expansion of CMV-specific T-cells. These cells can constitute a significant portion of the total T-cell population, particularly in older adults. CMV-specific CD8+ T-cells can become highly expanded, often dominating the CD8+ T-cell repertoire (29, 30). These cells are important for controlling viral replication through their cytotoxic functions. CMV-specific CD4+ T-cells are also expanded and play a critical role in supporting CD8+ T-cell responses and antibody production. CMV infection has been implicated in immunosenescence, the gradual deterioration of the immune system associated with aging. This is partly due to the chronic antigenic stimulation by CMV (31, 32). Over time, there is an accumulation of highly differentiated effector memory T-cells and effector T-cells, many of which are specific for CMV. The large clonal expansion of CMV-specific T-cells can reduce the diversity of the T-cell repertoire, potentially impairing the ability to respond to new infections or vaccinations (33). Despite their strong functional capabilities, CMV-specific T-cells can also express markers of cellular exhaustion and senescence, such as PD-1 and KLRG1, altering the broader immune regulatory environment (34, 35). Managing CMV-specific T-cell responses is crucial in SOT. CMV reactivation is controlled by cytotoxic CD8+ T-cells; in patients treated with immunosuppressants the immune control is ineffective and the stimulated CMV replication shifts towards clinically significant reactivation (36), so anti CMV prophylaxis is necessary, eventually in combination with anti-CMV immunoglobulins supplementation as suggested by several studies (37). CMV reactivation and disease associated with tissue injury represent a major risk factor for acute and chronic rejection, in part by promoting several other opportunistic infections (38). These data support the control of viraemia in recipients even in the absence of symptoms and the long-lasting Valganciclovir prophylaxis especially in CMV-positive donor and recipients.

### Other viral infections

A wide range of other viral infections also complicate LuTx. Among these, community-acquired respiratory viruses (CARV) (e.g., influenza virus, respiratory syncytial virus, adenovirus, parainfluenza virus, human metapneumovirus, rhinovirus etc.), herpes simplex virus and varicella-zoster virus are frequently responsible for lung allograft infection in LTR (39, 40). CARV infections in LTR have a high rate of progression to pneumonia and can be a trigger for immunologically mediated lung allograft injury (41, 42). Inflammation mediated by viral infections involves chemotactic cytokines, such as IL-1, TNF, IL-6, and IL-8, which recruit alloreactive leukocytes to the site of infection. This creates an environment that is conducive to immune-mediated injury and allograft rejection (43). Furthermore, respiratory viral infections post-LuTx induce circulating exosomes containing lung self-antigens, viral antigens, and 20S proteasome. These exosomes trigger immune responses to self-antigens, leading to CLAD as observed in immunized mice, highlighting a potential mechanism for increased rejection risk in transplant recipients with symptomatic viral infections (44). A literature review conducted by de Zwart and colleagues showed a high incidence of CLAD following infection with human metapneumovirus, influenza virus, and respiratory syncytial virus, despite an overall low incidence of 30-day mortality (45).

### Mycobacterial infections

Infection by Mycobacterium tuberculosis in SOT can be a reactivation of a primary infection, donor-transmitted, or a primary infection (46). Diagnosing pulmonary tuberculosis in SOT is difficult due to atypical clinical presentations and increased chances of false negativity during testing for disease (47–49). Studies have shown that rejection was more frequent in recipients with tuberculosis, and that both patient survival and graft survival times were shorter compared to recipients without tuberculosis (50, 51). Treatment challenges include interactions between immunosuppressive and antitubercular medications, allograft-related drug toxicities, and inadequate immune responses due to exogenous immunosuppression (52, 53). As for non-tuberculous mycobacteria (NTM), the incidence rates of infection in LTR range from 1.5% to 22.4% (54), with species from the Mycobacterium avium complex (MAC) being the most common findings, followed by Mycobacterium abscessus (14, 55). When treating NTM infection in LTR, generally a course of approximately one year of treatment following negative cultures is recommended, with adjustments for medication intolerance (56). Significant drug interactions, particularly with antimycobacterial agents like rifampin and clarithromycin, and immunosuppressive medications, further complicate treatment and can expose to the risk of lung allograft dysfunction (57). If possible, immunosuppression should be reduced, weighing the decision against the potential risk of organ rejection and allograft dysfunction. If immunosuppression reduction is not feasible or if there is a high disease burden (such as disseminated disease or smear-positive lung disease), prolonged therapy should be considered (58).

### Fungal infections

Invasive fungal infections usually occur within the first 3 to 12 months after LuTx (59) with an incidence of 8,6% in the first year after surgery (60). Aspergillus species and Candida species are the most common fungal pathogen observed and are responsible for the majority of infections that occur after transplant (61). Invasive fungal infections, particularly invasive aspergillosis, are linked to chronic allograft rejection (62). Aspergillus colonization, even without invasive infection, has also been linked to Bronchiolitis Obliterans (BOS) and BOS-related mortality, independent of rejection (63). Aspergillus colonization is linked to gene expression profiles that are involved in defense mechanisms, particularly cytokine signaling. The process of epithelial wounding, along with the innate immune response to chitin found in the fungal cell wall, may play crucial roles in connecting Aspergillus colonization to CLAD (64). As per other pathogens, allograft rejection and the consequent need for immune augmentation are a known risk factor for invasive fungal infections (65).

## Lung allograft rejection as a result of infection

Lung allograft rejection encompasses a spectrum of immune-mediated responses that compromise graft function. Rejection episodes after LuTx are defined by the nature of the prevailing immune response and can be divided into different categories depending on the immunological pattern of rejection (**Table 1**).

**Table 1:** Overview of the different forms of rejection in Lung Transplantation.

Form of Rejection	Description	Expected Timeframe	Physiopathologic Features
Acute Cellular Rejection (ACR)	T-cell mediated lung allograft damage.	Shortly after transplantation, with peak incidence during the first year.	Perivascular and peribronchiolar lymphocytic infiltration.
Antibody-Mediated Rejection (AMR)	Allograft dysfunction caused by circulating donor-specific antibodies, either pre-formed (hyperacute AMR) or newly-formed (late AMR)	- Hyperacute AMR: hour to days after transplantation - Late AMR: weeks to months after transplantation, contributes to chronic rejection over time.	Complement-driven endothelial damage, capillaritis and thrombosis, with evidence of circulating donor-specific antibodies.
Chronic Rejection (CLAD)	Chronic lung allograft dysfunction including several subtypes.	Develops in about 50% of lung transplant recipients within 5 years after transplantation.	Persistent low-grade T-cell and antibody responses cause chronic inflammation and fibrosis. Alloimmune responses and repeated injuries (e.g., infections) contribute.
Bronchiolitis Obliterans Syndrome (BOS)	Subtype of CLAD characterized by airflow obstruction.		Lymphocytic bronchiolitis, fibrotic obliteration of small airways.
Restrictive Allograft Syndrome (RAS)	Subtype of CLAD characterized by restrictive ventilatory defect and ground glass opacities on lung CT scan.		Diffuse alveolar damage, fibrosis, thickened alveolar walls.

Infection is a critical factor in triggering lung allograft rejection due to its impact on the recipient’s immune system. Reducing the risk of rejection while maintaining immune competence towards infection can be achieved through a better understanding of lung allograft immune tolerance. Key players include T regulatory lymphocytes (Treg), which suppress effector T cells that target donor major histocompatibility complex (MHC) molecules, thereby promoting graft acceptance (66, 67). Additionally, immune checkpoint molecules such as PD-1/PD-L1 are crucial in regulating T cell activation thresholds, balancing immune tolerance and activation (68). Experimental models have demonstrated that manipulating costimulatory pathways, such as B7-CD28 and CD40-CD40L interactions, can induce long-term tolerance of lung allografts by preventing full T cell activation and subsequent immune-mediated damage (69). Clinical strategies leveraging donor bone marrow infusion have shown promise in promoting donor-specific hyporeactivity, potentially allowing for reduced immunosuppression post-transplantation while maintaining graft integrity (70).

### Acute cellular rejection

ACR is a T-cell mediated organ damage commonly found shortly after LuTx, with 34% of the cases occurring in the first year post-LuTx (71). While most episodes of ACR respond to first-line immunosuppressive treatment (72), it constitutes one of the main risk factors for subsequent CLAD development (73, 74). Infection of the lung allograft is a major risk factor for ACR due to the exposure of donor antigens from epithelial injury, leading to allo-sensitization (75). In addition to pulmonary infection, non-immunological processes like ischemia-reperfusion injury can activate local innate immunity, leading to acute rejection (76). Interestingly, in recent years, changes in the lung microbiome, particularly microbial dysbiosis and the enrichment of certain bacteria, have been linked to post-transplant complications. In particular, dynamic shifts in microbial diversity and taxonomic trajectories in the lower airway have been associated with ACR, suggesting that microbial signatures could serve as potential biomarkers for rejection risk (77, 78). Conversely, risk factors for infections occurring more than six months post-transplant include early acute rejection, recurrent CMV infection, and prior bacterial infections (24, 79).

### Antibody-mediated rejection

AMR in LuTx is caused by donor-specific antibodies (DSA) directed against human leucocyte antigens (HLA) expressed on the donor lung (80), triggering the complement cascade and causing acute injury. This may lead to hyperacute rejection, characterized by graft thrombosis and necrosis, often resulting in short-term poor prognosis (81, 82). Advances in HLA antibody detection prior to transplantation have enabled virtual cross-matching, reducing the incidence of hyperacute rejection by ensuring donor-recipient compatibility (83). On the other hand, AMR is currently recognized as a relevant form of graft rejection beyond the immediate post-transplant period. In this setting, AMR is driven by newly-formed DSA that target endothelial cells, leading to capillaritis and microvascular injury over time. Lung allograft infections can exacerbate this process by causing tissue injury, which exposes donor antigens to the recipient’s immune system, thereby stimulating the production of DSA (25). Additionally, epithelial damage caused by these bacteria results in increased HLA-DR expression and soluble HLA class I release, further promoting alloreactive lymphocyte activation (15). Notably, more than half of LTR develop *de novo* DSA within three months post-transplantation (84). As literature suggests that AMR might promote the development of CLAD (85–87), many authors warrant strict DSA screening after LuTx and favor the use of preemptive antibody-directed therapy (88–90).

### Chronic rejection

Chronic rejection is a definition that includes different variants of chronic dysfunction of the lung allograft grouped under the term Chronic Lung Allograft Dysfunction (CLAD). CLAD usually results in permanent lung allograft damage and ultimately in the deterioration of lung function (91). It has at least four different subtypes: Bronchiolitis Obliterans Syndrome (BOS) (70% of cases), Restrictive Allograft Syndrome (RAS), mixed forms and undefined phenotypes (92). As mentioned above, both ACR and AMR increase the risk of developing CLAD (93, 94). Immunological pathways leading to CLAD and more specifically BOS, are significantly influenced by infection and the resulting inflammatory responses. For example, *Pseudomonas aeruginosa* and *Staphylococcus aureus* are key pathogens that interact with chemokine receptors, notably CXCR1/2, leading to increased recruitment of neutrophils and lymphocytes into the lung allograft. This recruitment is driven by ELR+ chemokines such as CXCL1, CXCL5, and CXCL8, which are elevated in response to these infections (15). More generally, respiratory infections, regardless of pathogen type, strongly expose to the risk of CLAD development, with a single infection episode increasing CLAD occurrence significantly. In a study by Shino et al., the severity of the immune response, indicated by elevated bronchoalveolar lavage fluid CXCL9 levels during infection, correlated with higher CLAD risk, highlighting a dose-response relationship (95).

## Immunosuppression in lung transplantation and the risk of infection

**Figure 3:**
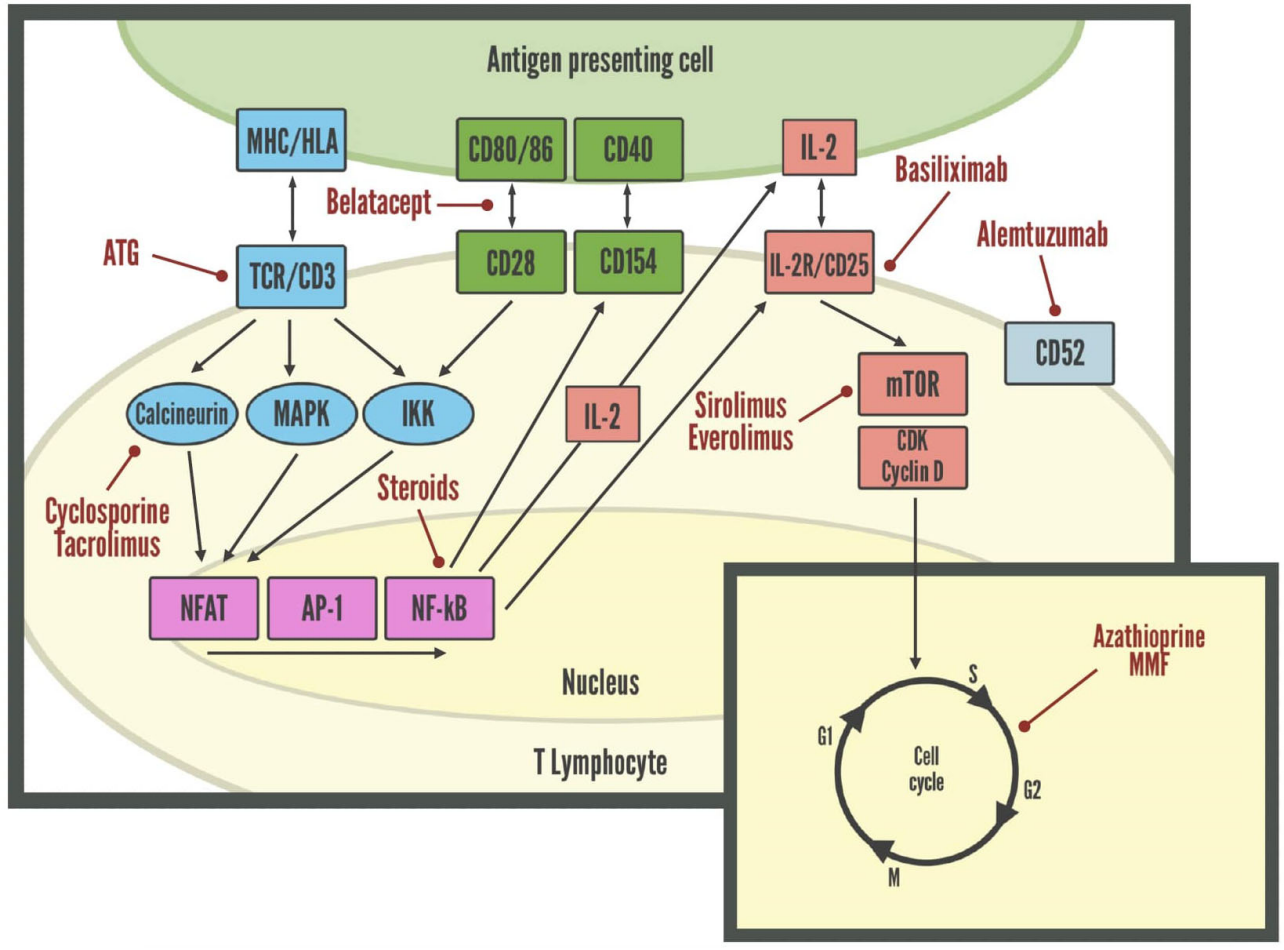
Molecular targets of immunosuppressive drugs in lymphocytes. AP-1, Activator protein 1; ATG, Anti-thymocyte globulin; CDK, Cyclin-dependent kinases; HLA, Human leukocyte antigen; IKK, Inhibitor of nuclear factor-κB kinase; MAPK, Mitogen-activated protein kinase; MHC, Major histocompatibility complex; mTOR, Mammalian target of rapamycin; NFAT, Nuclear factor of activated T-cells; NF-kB, Nuclear factor kappa-light-chain-enhancer of activated B cells; TCR, T-cell receptor.

Risk of rejection is highest earlier after LuTx, thus requiring stronger immunosuppression immediately post-transplant, differently from maintenance immunosuppressive therapy which becomes less intense over time (96). Immunosuppressive agents may lead to adverse effects, including drug-induced toxicity and opportunistic infections (97). Although immunosuppressive protocols vary from center to center, most of them use induction therapy, which is given peri-operatively to reduce the risk of acute rejection, and it consist in polyclonal antibody preparations such as antithymocyte globulin (ATG), alemtuzumab, interleukin 2 receptor antagonists (IL2RAs) as basiliximab, or tocilizumab (8, 97, 98). Conventional maintenance therapy consists of triple-drug therapy with a calcineurin inhibitor such as cyclosporine or tacrolimus, an antiproliferative agent such as azathioprine, mycophenolate, sirolimus, everolimus, and a corticosteroid (8). The most common regimen both at 1- and 5-years’ follow-up is tacrolimus, mycophenolate, and prednisone, though it is not uncommon for patients to require the switch to alternative immunosuppressive regimens based on individual tolerability (99). A graphical summary of the main mechanisms of action of these drugs is provided in **Figure 3**.

### Antithymocyte globulin

ATG is a polyclonal antibody resulting in a dose-dependent depletion of T cells both in the periphery and secondary lymphoid organs. It also causes CD20+ cells (B cells) and CD16+/56+ cells (NK cells) levels to decrease (100). Immunosuppression induction with ATG is associated with a higher risk of infections, as documented in LTR developing coronavirus disease-2019 (COVID-19), *Enterococcus* infection, or *Klebsiella* pneumonia (101). This effect is augmented when ATG is combined with other immunosuppressant. Induction therapy with ATG, along with steroid maintenance, high calcineurin inhibitors doses, and CMV infection is associated with late-occurring pneumonia in post-transplant patients (102). Infectious risks are heightened when antilymphocyte therapies are used for treating graft rejection compared to induction immunosuppression, warranting appropriate monitoring and prophylaxis for *Pneumocystis carinii* pneumonia, CMV, and fungal infections, along with EBV and BKV monitoring (103).

### Alemtuzumab

Alemtuzumab is a humanized monoclonal antibody leading to profound depletion of T cells, and to a lesser degree B cells and monocytes. Notably, this drug was also shown to induce the proliferation of Treg cells (99, 104, 105). Alemtuzumab-treated patients were reported to experience the lowest rate of infection in the first year after transplantation (106). Notably, only limited alemtuzumab dosing appears safe and effective for induction therapy in SOT, as extended alemtuzumab exposure combined with steroid and calcineurin maintenance therapy is instead associated with a higher risk of infectious (bacterial, fungal and viral) complications and ACR (107).

### Basiliximab

Basiliximab is an interleukin-2 receptor antagonist (IL-2 RA) used in more conservative induction immunosuppression regimens (108). It is a monoclonal antibody directed against the IL-2 receptor α-chain expressed on activated T cells. The rate of serious infectious adverse events in SOT receivers appears lower in those treated with basiliximab compared with alemtuzumab or ATG (99). Induction therapy with ATG, and T-cell depleting agents is on the other hand associated with a greater incidence of CMV, Epstein-Barr virus, BK polyomavirus infections, and interstitial pneumonia, and a higher risk of bacterial infections compared with IL–2a receptor antagonists (103, 109).

### Tocilizumab

Tocilizumab is an IL-6 inhibitor, showing promise in the treatment of AMR in renal transplant recipients. Excessive IL-6 production is associated with activation of T-helper 17 cells and inhibition of Treg, modulating several immune pathways responsible for allograft injury. This suggests that anti-IL-6/IL-6R blockade could be effective in modifying T- and B-cell responses to induce desensitization and prevention and treatment specifically of AMR (110). The use of tocilizumab in LuTx has shown to be able to induce a better clearance of DSA, lower recurrence of DSA, lower incidence of *de-novo* DSA, and lower rates of graft failure (98). The ALL IN LUNG study (clinicaltrials.gov - NCT06033196) is currently exploring the hypothesis that treatment with triple maintenance immunosuppression plus Tocilizumab is superior to triple maintenance immunosuppression alone.

### Belatacept

Belatacept is a fusion protein composed of the modified extracellular domain of cytotoxic T lymphocyte-associated protein 4 (CTLA-4) and the Fc domain of human immunoglobulin IgG14 (8). It blocks co-stimulation by binding to CD80 and CD86 receptors on Antigen Presenting Cells (APCs). This eventually prevents binding of CD28 on the T cell. Recently, few cases of Pneumocystis jirovecii-related pneumonia have been documented under belatacept and everolimus immunosuppressant regimen, highlighting the importance of pneumonia prophylaxis after conversion to belatacept (111).

### Calcineurin inhibitors

Both cyclosporine and tacrolimus inhibit the phosphatase activity of calcineurin, ultimately reducing cytokine production (mostly IL-2) and inhibiting T cell activation. As reported by a clinical trial of tacrolimus *vs*. cyclosporine in LuTx, the overall incidence of infections appears similar, although bacterial infections were more frequent with cyclosporine, whereas fungal infections were more common with tacrolimus (112). As documented by a more recent study, cyclosporine may be a risk factor for the development of tuberculosis among renal transplant recipients (113). Increasing evidence indicates a relatively low risk of viral infections in patients receiving cyclosporine as this drug appears to inhibit the replication of some viruses (114). However, the mechanisms underlying cyclosporine-induced antiviral activity remain to be confirmed and elucidated.

### Anti-proliferative agents

Azathioprine reduces T cell proliferation whereas mycophenolate results in inhibition of T and B cell proliferation by blocking DNA synthesis (115). Opportunistic infections frequently occur in post-transplant patients treated with these drugs, a possible consequence of their strong hematologic adverse effects, such as leukopenia, and neutropenia (116). Mycophenolate was associated with increased risk for tissue-invasive CMV, herpes simplex virus, varicella-zoster virus, and Aspergillus infections (117, 118). Delayed severe pneumonia has also been reported following mycophenolate plus corticosteroids in patients with autoimmune diseases (119). Sirolimus and Everolimus are endowed with very broad immunological effects that include the generation and expansion of Treg as well as the maturation and function of DC (120). Mammalian target of rapamycin (mTOR) inhibitors, such as sirolimus and everolimus, have shown potential anti-CMV effects by blocking the mTOR pathway, which is crucial for viral replication (121). By inhibiting this pathway, mTOR inhibitors reduce CMV replication and may decrease the incidence and severity of CMV infections (122).

### Corticosteroids

Prednisone, Prednisolone, Methylprednisolone, and Dexamethasone are the glucocorticoids most used in immunosuppressive regimens for LuTx. They upregulate transcription of anti-inflammatory genes but downregulate that of inflammatory genes (123). Long-term corticosteroids use is associated with several side effects including opportunistic infections, especially when administered concomitantly with other immunosuppressive agents (123, 124). In line with this, corticosteroids’ use appears to increase susceptibility to invasive fungal and viral infections, as well as tuberculosis, in transplant recipients (123, 125). Notably, severe metabolic complications are often the result of long-term corticosteroid use. Thus, the institution of a steroid-free immunosuppressive regimen is highly desirable. Late steroid withdrawal appears safe in stable patients after LuTx, as evidenced by the lack of rejection or deterioration in pulmonary function along with amelioration of lipid profile and blood pressure over a median follow-up of 19 months (126).

The use of immunosuppressive drugs in LuTx is essential to prevent rejection but comes with a significant trade-off in increased susceptibility to various infections. Understanding the specific mechanisms of these drugs helps in tailoring prophylactic and therapeutic strategies to mitigate infection risks while maintaining adequate immunosuppression to protect the transplanted organ. Regular monitoring, prophylactic antimicrobial treatments, and prompt management of infections are crucial components of post-transplant care.

## Conclusion

LuTx is a life-saving therapeutic option for patients with end-stage lung disease. Despite significant advancements in surgical techniques and overall patient care, long-term outcomes post-LuTx are considerably worse than other SOT, largely due to lung allograft rejection, which still to date remains a relevant problem. The management of LTR requires a delicate balance between proper immunosuppression to prevent rejection and appropriate host competence in an allograft that is exposed to environmental pathogens and pollutants. Current immunosuppressive regimens inhibit multiple immune pathways compromising the host’s defense against infections that are one of the major causes of post-LuTx morbidity and mortality. This broad-spectrum immunosuppression currently used in LTR stems from a limited understanding of the precise mechanisms underlying rejection, both acute and chronic. Future research should also aim to identify diagnostic and prognostic markers of organ tolerance, which could assist clinicians in distinguishing between rejection and infection, improving graft prognosis. This knowledge could facilitate the identification of biomarkers for early differential diagnosis post-LuTx and enable personalized therapeutic strategies. The side effects of immunosuppressive therapy, infections and CLAD remain important challenges impairing long-term survival. Advances in prevention and treatment of chronic rejection are critical to further improve outcome. Investigations into the optimal level of immunosuppression that safeguards the allograft while preventing opportunistic infections are also essential. As our understanding of these complex interactions expands, we anticipate that patient quality of life and outcomes will continue to improve.
